# AV loop free flap: an interdisciplinary approach for perineal and sacral defect reconstruction after radical oncological exenteration and radiation in a colorectal cancer patient

**DOI:** 10.1186/s12957-019-1698-1

**Published:** 2019-09-02

**Authors:** Jan Matschke, Rafael Armbruster, Christian Reeps, Jürgen Weitz, Adrian Dragu

**Affiliations:** 1 Department of Plastic and Hand Surgery, Faculty of Medicine and University Hospital Carl Gustav Carus, TU Dresden, Dresden, Germany; 2 Department of Visceral, Thoracic and Vascular Surgery, Faculty of Medicine and University Hospital Carl Gustav Carus, TU Dresden, Dresden, Germany

**Keywords:** Rectal cancer, Local recurrence, Interdisciplinary approach, Free latissimus dorsi flap, Arterio-venous loop, Two-time approach

## Abstract

**Background:**

The free flap transfer of a latissimus dorsi flap (LDF) for the closure of sacral wound defects after pelvic exenteration and radiation therapy offers a successful tool of the plastic surgeon. This case report shows the successful coverage using an upstream arterio-venous (AV) loop in combination with an LDF.

**Case presentation:**

We describe the case of a patient who underwent a pelvic exenteration and radiation therapy due to a local recurrence of rectal cancer. The initially used VRAM flap could not ensure a satisfactorily wound closure. An interdisciplinary approach first yielded an AV loop using both greater saphenous veins and was connected to the arteria and vena femoris followed by a free LDF transfer, which was performed 11 days later. The result was an excellent reconstructive and plastic coverage of the sacral wound defect with a well-perfused LDF. The long-term result showed a perfectly integrated flap in the sacral region.

**Conclusion:**

We recommend the free LDF for the coverage of large wound defects in irradiated areas after the failure of VRAM flap. If an AV loop is necessary within the flap transfer, we recommend conducting two procedures to guarantee the perfusion of the AV loop.

## Background

We present a successful interdisciplinary reconstructive case of wound closure of a wide sacral wound defect using a free latissimus dorsi flap (LDF) in combination with an arterio-venous loop (AV) as the perfusion conduit. Prior to plastic reconstruction, the patient underwent multiple surgeries and pelvic exenteration suffering from a colorectal cancer recurrence in an advanced stage.

Sacral wound defects are a common complication after sacrectomy and radical pelvic exenteration in patients with gynecologic, urologic, or gastrointestinal cancer.

Due to the poor local soft tissue quality after radiation therapy, the surgical management of such complex wounds and soft tissue defects is very challenging. The surrounding tissue perfusion is compromised and especially the local wound edges are highly pathologically altered; thus, local flaps or even primary or secondary wound closure strategies are not promising or even contraindicated.

For that reason, free flaps are an essential reconstructive tool in post oncological coverage of radiation-induced wound defects.

In these types of wounds, it is usually impossible to accomplish a tension-free coverage because of the large distance between the wound edges of the defect. Even in cases in which a small defect size would allow a primary or secondary wound closure, the problem of the irradiated wound edges that are sutured together remains. In addition, every reconstructive plan of a free flap needs adequate and close to the defect recipient blood vessels in order to perform the microvascular anastomosis. Especially in this highly morbid patient group, there are usually no adequate and unradiated local recipient vessels.

In these cases, an AV loop facilitates the approximation of recipient blood vessels close to the wound defect. This additional procedure represents an important technical tool as a first step of a reconstructive plan in soft tissue defect coverage using a free LDF in extended post oncological sacral wound defects.

## Case presentation

We present a 65-year-old male with reduced general state of health. The patient had been first diagnosed with rectal cancer in March 2014 (pT3 N0 cM0 G1 R0). Consequently, he received neoadjuvant radiation therapy with a low anterior rectal resection with formation of a protective ileostomy in July 2014 which was subsequently taken down. During a colonoscopy in May 2017, a local recurrence of the rectal cancer was detected and histologically confirmed. A PET-MRI scan proofed a presacral rectal cancer recurrence with infiltration of the Os sacrum (sacral vertebrae 3-5) without distant disease. The staging was rpT4b, rpN0, rpM0, L0, V1, Pn1, and Gx.

Within an interdisciplinary tumor board in January 2018, the decision to perform the total pelvic exenteration and radical tumor resection was taken. The surgery was performed in February 2018. For reconstruction of the sacral wound defect in the perineal area, a pedicled vertical rectus abdominis myocutaneous (VRAM) flap was used.

Postoperative histologic examination of the specimen confirmed complete tumor resection. In the further clinical course, the patient developed a sacral wound healing complication in the radiated perineal area which was managed by multiple debridements and VAC therapies.

**Fig. 1:**
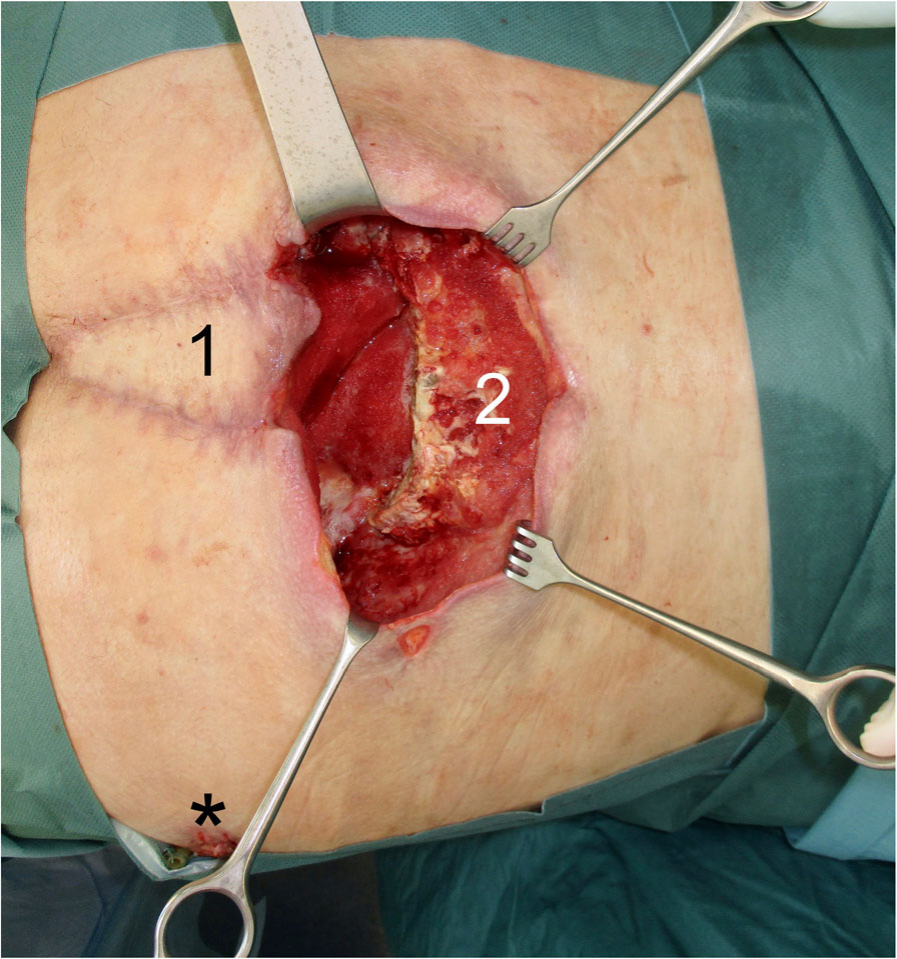
The sacral wound defect after the pelvic exenteration. (1) VRAM flap. (2) Sacral wound defect. Asterisk indicates the position of the vertex of the AV loop

After interdisciplinary reevaluation, it was decided to perform a free LDF to cover the 23 × 18 cm wound defect (Fig. 1), as local measures were not successful.

Due to the lack of adequate local recipient blood vessels close to the wound defect, an AV loop using both greater saphenous veins was formed and tunneled subcutaneously prior to the coverage with the free LDF.

**Fig. 2:**
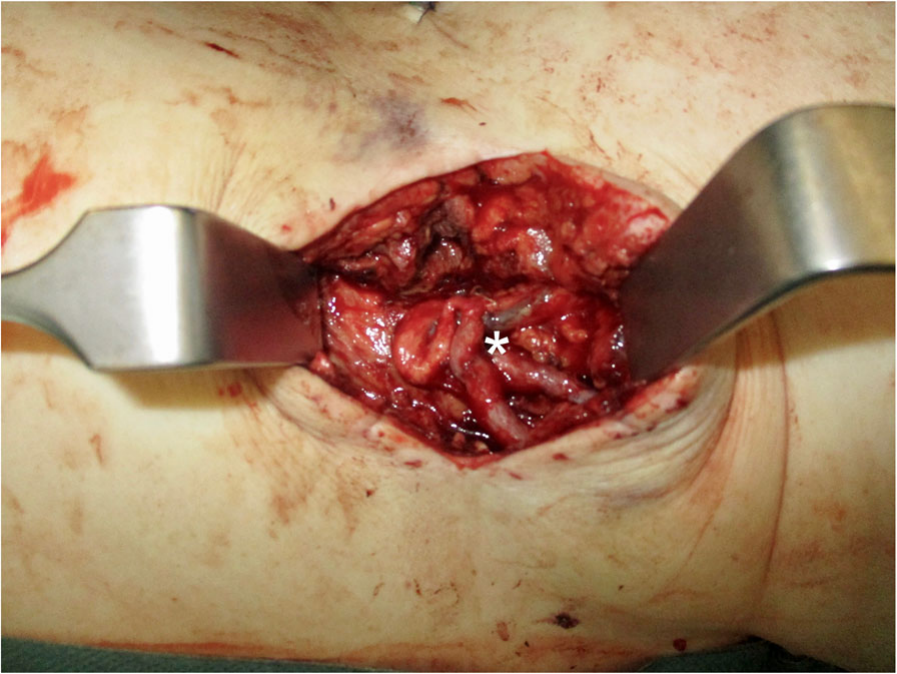
The vertex of the AV loop above the iliac crest. Asterisk indicates the AV loop as recipient vessels and anastomosed pedicle-vessels of the LDF

Both greater saphenous veins were anastomosed in end-to-side technique to the left arteria and vena femoris. The vertex of the AV Loop was placed above the iliac crest in the middle of the axillary line (Fig. 2).

After ensuring the blood flow through the AV loop over a period of 11 days, the reconstructive procedure with the free LDF was performed in June 2018. First, another radical debridement of the sacral wound defect was done.

The flap was carefully microsurgically dissected, and special care and planning was taken to the exact position of the skin island on the flap. The size of the skin island was planned to match the size of the sacral wound defect.

**Fig. 3:**
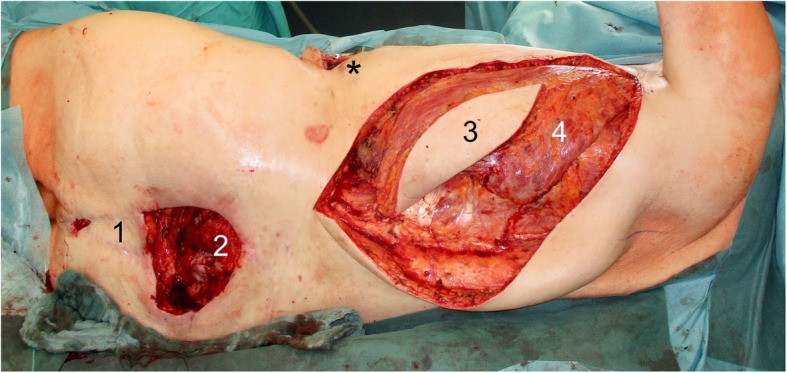
Intraoperative findings during the LDF transfer. (1) VRAM flap. (2) Sacral wound defect. (3) Skin island of the LDF. (4) Musculus latissimus dorsi. Asterisk indicates the position of the vertex of the AV loop

**Fig. 4:**
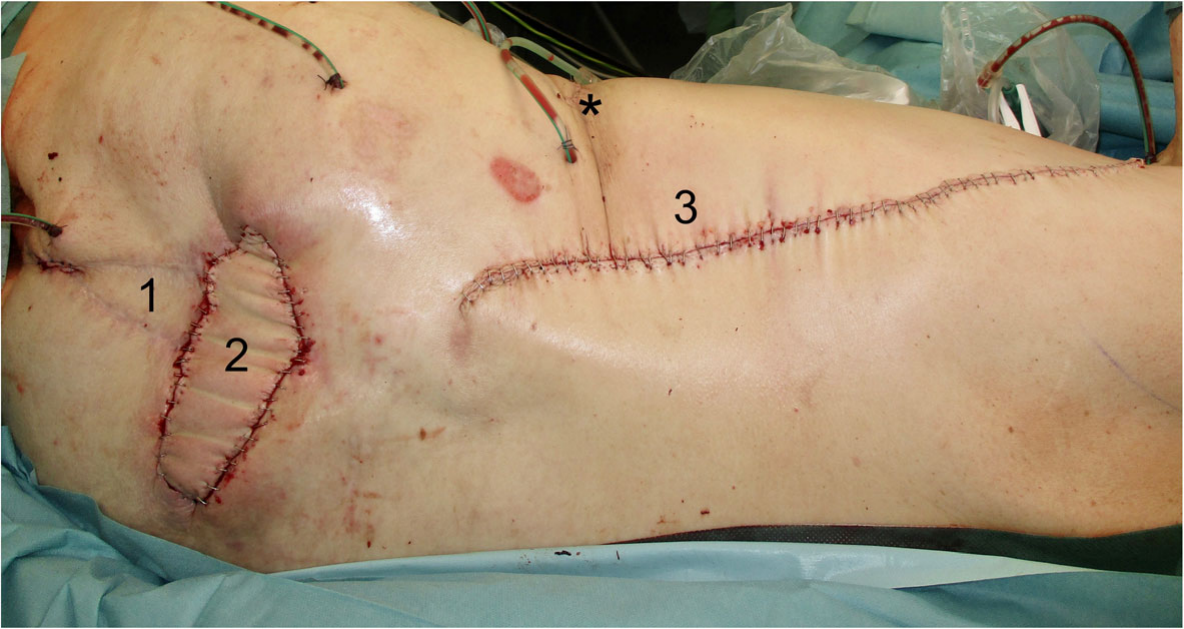
Immediate postoperative result with well-perfused LDF. (1) VRAM flap. (2) Closed sacral wound with skin island of the LDF. (3) Closed donor side of the LDF. Asterisk indicates the position of the vertex of the AV loop

The surgical incision was placed right above the AV loop, which showed an excellent blood flow. The LDF’s vascular pedicle, the arteria, and vena thoracodorsalis were exposed. The following dissection of the flap was performed without difficulty. The size of the skin island was 20 × 15 cm (Fig. 3). After the microvascular dissection of the vascular pedicle of the LDF and the division of the AV loop followed by the identification of the venous and arterial pedicle, the venous anastomosis was followed by the arterial anastomosis. The ischemia time of the flap was 78 min. Finally, we achieved an excellent reconstructive and plastic coverage of the sacral wound defect with a well-perfused LDF (Fig. 4).

**Fig. 5:**
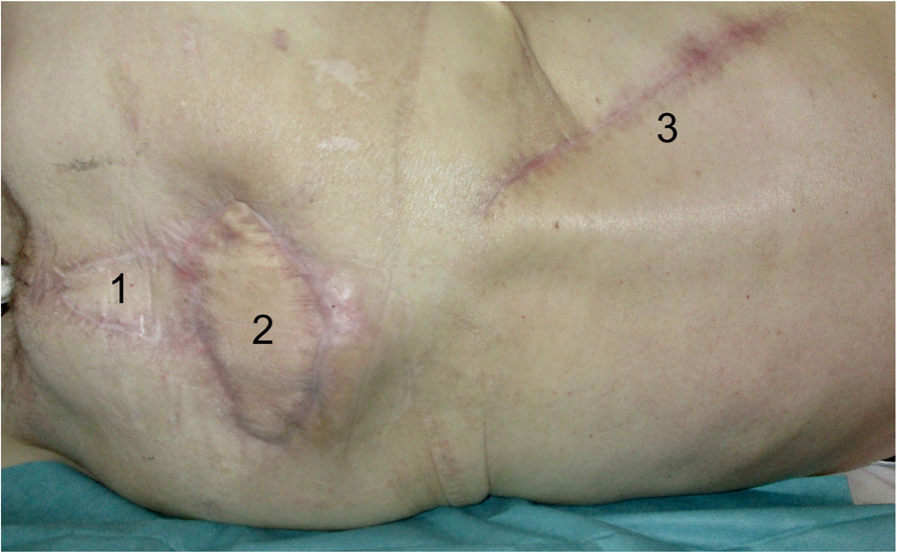
Thirteen-week long-term result with stable flap integration. (1) VRAM flap. (2) Healed LDF in the prior sacral wound defect. (3) Scar of the donor side of the LDF

**Fig. 6:**
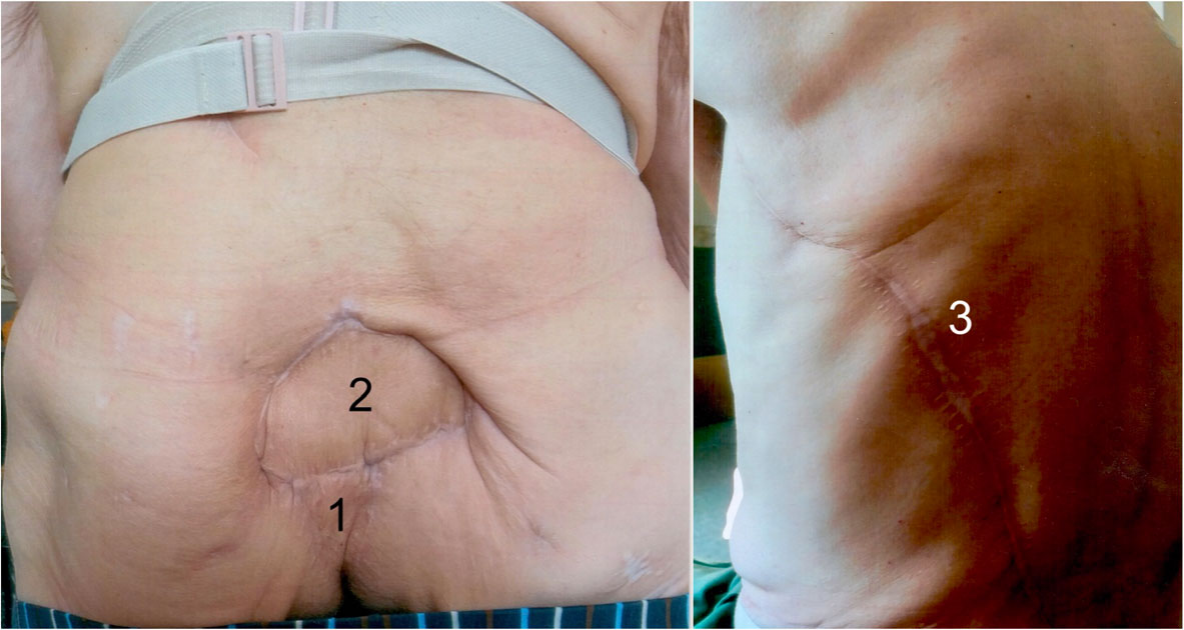
One-year long-term result with stable flap integration. (1) VRAM flap. (2) Healed LDF in the prior sacral wound defect. (3) Scar of the donor side of the LDF

The patient was dismissed from hospital in August 2018. In the follow-up time, perfect and stable flap integration within the previous sacral soft tissue defect was observed (Figs. 5 and 6).

## Discussions and conclusions

Due to its complexity, a pelvic exenteration is correlated with a complication rate up to 50%. The most common complications are postoperative wound infections [1]. The consequences include postoperative, major sacral, considerable wound defects.

In 95% of the cases, tissue defects occur within and around the fields of the radiation therapy. Damages include mild to severe erythema and ulcerations. Because of the irreversible tissue damage due to reduced tissue perfusion and fibrotic remodeling, the tissue only offers a reduced resistance against infections [2]. In addition, a further major problem is wound healing problems and seroma. This issue represents especially a challenge for plastic surgeons as the previous performed radical oncologic tumor resections within radiated tissue leave deep and big tissue defects.

The damaged tissue does not offer ideal circumstances for a local flap due to its lowered susceptibility to infection and reduced perfusion. In radiated tissue, the free flap is the best opportunity and superior to the local flap [3]. Primary or secondary direct wound closure is even contraindicated as a superficial skin closure under tension with both wound edges being radiated is no adequate reconstructive concept.

The benefit of using free flaps lies in the not radiated origin of the free flap, which reduces the occurrence of impaired wound healing. The prerequisite for a free flap is a sufficient vascular supply in the wound defect. This is often absent due to radiation therapy or due to the new vascular anatomy after radical oncologic resection.

An AV loop offers the attractive opportunity to approximate donor blood vessels to the wound defect. This facilitates a tension-free coverage of the wound defect. An AV loop enables the bridging between recipient and donor blood vessels, if the distance of these is too large. In a trial published by Meyer et al., complications occurred in half of the AV loop operations combined with a free flap. Nevertheless, most flaps (85%) could be saved [4, 5]. Despite a high rate of early complications, the combined treatment using an AV Loop and a free flap offers a superb treatment option [6].

The usual approach includes two operations as a two-stage reconstructive concept. During the first operation, the AV loop is placed. During the second operation, the free flap transfer is performed. The other strategy implies the AV loop formation and the free flap transfer within one-step operation; however, data and clinical experience show that the complication rates are higher. Nevertheless, there is also data which controversially debates whether to perform one or two procedures [7, 8].

The main advantage in performing the surgery in two sessions is the opportunity to ensure the perfusion of the AV loop before raising the flap. This prevents flap loss due to a malfunctional AV loop [9].

Other centers prefer the one-step surgery. They argue with the absence of the significant benefit of the two-step approach and the general risk of another operation.

Generally, it should be considered individually which strategy to perform. In some cases, a two-step approach might not be possible due to intraoperative complications. In case of a short and low risk AV loop, a two-time approach might not be justified [10].

The free LDF is mostly raised as a large volume myocutaneous flap with its anatomically defined vascular pedicle. Due to its universal applicability, the LDF is considered as the workhorse in reconstructive plastic surgery especially for deep and large soft tissue defects. It can be applied for thoracic defects as well as wound defects on the limbs [11].

The benefits of the LDF include its constant anatomy, which allows a standardized approach.

Given its large volume, the LDF enables the coverage of extended defects. As in our presented case, an area of 23 × 18 cm was covered. It fills up defects in the lesser pelvis after radical pelvic exenteration and prevents herniation of abdominal organs and enables further radiation therapy.

In some special cases though, a new way of wound reconstruction using an LDF publicized. It is possible to use a reverse pedicled LDF. A clinical study from 2019 showed the possible use of a reversed LDF to cover defects in the sacral region [12]. In our presented case, it might have been an eventuality to close the sacral sound defect using a bilateral reversed LDF. An advantage of the reverse LDF would be the omission of an AV loop, which would lower the complexity of the surgery.

Another possibility for wound closure in the cavities in the perineal region could be an anterolateral thigh flap (ALTF) [13]. The main advantage would be the pedicle which makes a free flap transfer redundant. In our case, as stated above, the defect would have been out of reach for this special technique. In some cases, the ALTF is considerable and offers a keyhole incision which is very useful in defects in the perianal region for the recovery for the tissue around the cavity.

We recommend the free LDF for the coverage of large wound defects in irradiated areas. If an

AV loop is necessary within the flap transfer, we recommend conducting two procedures to guarantee the perfusion of the AV loop. The main advantages of the free LDF include the large volume and the standardized raising and a low complication rate. In the presented case, the plastic-reconstructive coverage complemented the radical oncologic therapy and avoided the hospitalization of the patient. It allowed a life within the familiar surroundings for the patient.

## Data Availability

The datasets used and/or analyzed during the current study are available from the corresponding author on reasonable request.

## Abbreviations

ALTF: Anterolateral thigh flap
AV: Arterio-venous
LDF: Latissimus dorsi flap
VRAM: Vertical rectus abdominis myocutaneous
